# A Clear Solution for Dirty Water

**DOI:** 10.1289/ehp.114-a424

**Published:** 2006-07

**Authors:** Tim Lougheed

Turning water into wine may be among the most venerable of miracles, but for Greg Allgood, the real miracle has been turning dirty water into drinkable water. He once wowed an audience in a Malawi village, where hundreds of inhabitants along with the country’s Minister of Health watched him transform a sample of the only local source of drinking water. “There were gasps of excitement when the water turned from this horrible, muddy dark color to crystal clear and safe,” he recalls.

Allgood was demonstrating PUR^™^, a modest-looking packet of powder that quickly turns turbid, health-threatening water into the kind of liquid most of us would pay to drink out of a bottle. PUR was developed in the late 1990s by household products giant Procter & Gamble (P&G) and shares its name—but not its technology—with home tap water filters sold by that company in developed nations. Now PUR occupies a place at the forefront of P&G’s Children’s Safe Drinking Water Program, a philanthropic initiative that Allgood directs.

Allgood spends about a third of his time in places like Malawi where people have limited or no access to treated, potable water sources. Worldwide, as many as 2 billion people drink water extracted from shallow wells or polluted lakes and rivers, with nothing like the municipal treatment systems that are taken for granted in most of North America and Europe. In the few developing locales where such infrastructure might exist—and indeed, even in the richest nations on the planet—this resource can be ruined suddenly by a natural disaster like a hurricane, earthquake, or tsunami, creating an immediate, desperate, and widespread need for safe drinking water.

## The Stuff of Life

Water can be the key to keeping death and disease at bay. Hydration is fundamental to bodily functions, including the ability to retain nutrients. Infants, the elderly, and immunocompromised persons are especially vulnerable to dehydration caused by diarrhea, which is in turn spawned by bacteria or viruses acquired from tainted drinking water. In African countries ravaged by HIV/AIDS, large portions of the adult population could likewise succumb to even limited numbers of parasites found in relatively clean water. “While [a healthy person] might take a couple of weeks to get over *Giardia*, it could be fatal to a person that has a reduced immune system,” says Allgood. As opposed to dealing with these ailments once they appear, purifying water can keep them from appearing at all.

The CDC became interested in point-of-use treatment when cholera exploded in Peru in 1991 and spread rapidly throughout Latin America. A dependence on questionable drinking water lay at the heart of this epidemic, and the Pan American Health Organization estimated that it would take some $200 billion and more than a decade to install the necessary municipal infrastructure to alleviate the problem throughout the region. The CDC sought alternatives to help affected populations in the meantime.

Chlorine bleach was among the most widely available disinfectants, although people had difficulty gauging how much was needed to treat a given amount of water without creating an unpleasant taste or harmful concentrations. The agency therefore supported development of special bottles of dilute bleach—the bottle caps were designed to hold just the right amount of solution to safely treat one jerry can of water.

These efforts caught the attention of P&G, the leading manufacturer of bleach in many of the affected countries. But while this approach continues to be used in many parts of the world, it does not remove suspended material from the water, leaving users with water that is microbe-free but can still look dirty. So in the mid-1990s, P&G struck a formal Cooperative Research and Development Agreement with the CDC, focusing on how drinking water could be even better treated at the point of use.

## Floccing Toward Solutions

P&G researchers tackled the challenge with flocculants, agents that promote molecular aggregation and can cause colloids or loose particles in a liquid to amass in clumps that sink to the bottom. Combined with large-particle calcium hypochlorite—essentially, powdered bleach—the result was PUR, a proprietary formulation that Allgood describes as reverse-engineering the municipal water treatment process.

Using PUR is like making a batch of powdered soft drink mix. Each packet of powder is designed to treat 10 liters of water. One simply tears open the packet, pours the powder directly into the water, and stirs. Within a matter of seconds, any floating material will start to flocculate into clumps that sink to the bottom. In no more than five minutes, all of the water is clear, and after standing for about 20 minutes, it will be completely disinfected. If desired, the solid remnants can be removed with the most basic of filters, such as a simple piece of cloth.

“The large particle size makes [the powder] slowly dissolve, so in essence it acts like a time-released formula of chlorine disinfectant,” Allgood says. “That’s important, because this product is meant to treat a huge range of waters, from clear to extremely contaminated.”

Even seasoned observers, including the scientists who initially refined and tested PUR, agree that its action is nothing less than dramatic.

“It was extremely impressive, and the most impressive thing about it was its simplicity,” notes John Perry, a microbiologist at Freeman Hospital in Newcastle upon Tyne, United Kingdom. He and his colleagues spent two years working closely with P&G, putting PUR through its paces in the laboratory.

“We would take a bucket of clean water and contaminate it with all sorts of things—lots of different types of bacteria, but also viruses, protozoan cysts, and they’d also put a lot of soil in it to mimic the kind of conditions that you get in the field,” Perry says. “We did a very detailed analysis of what came out at the end of the process, and all of these bacteria, viruses, and cysts had magically disappeared.”

These results were recounted in a paper coauthored by Perry that appeared in the June 2003 issue of the *Journal of Water and Health*. Other investigators have also published findings from applications of PUR in various settings, ranging from ongoing rural development activities in Kenya and Guatemala to crises like that in Haiti following Tropical Storm Jeanne in September 2004. Just a few months after Jeanne struck, various aid agencies purchased 13 million packets of PUR and transported them to parts of Sri Lanka, Indonesia, and the Maldives when they were struck by the great tsunami of December 2004.

## One Option of Many

In addition to its humanitarian value in disaster relief, the product is also being marketed as a household commodity in many other parts of the world where large portions of the population lack reliable water treatment. The pricing of such a good varies widely from one market to another, based on what the local market will be thought to bear. Sally Cowal, a senior vice president with the Washington, DC–based nonprofit firm Population Services International (PSI), oversees the complex dynamics of advertising and selling PUR in different countries.

“Because we’re in social marketing, we have a great belief that if you pay for something, you’re much more likely to use it than if it’s handed to you,” she says. Of PSI’s alliance with P&G, she says, “We’re learning a lot from one another. They don’t know particularly well how to reach the bottom of the pyramid in the countries we work in; that’s what we know really well. But they know things about brands and brand management and sophisticated marketing and sales techniques that we [can] learn from them.”

Neither of these organizations present PUR as a single, definitive answer to water treatment under any and all circumstances. Eric Mintz, chief of the Diarrheal Diseases Epidemiology Section of the CDC’s Foodborne and Diarrheal Diseases Branch, points out that dilute bleach, membrane filters, and solar (ultraviolet) disinfection each have their appropriate niche.

“We think those all have a place, and they all have advantages and disadvantages,” Mintz says. “Allowing people to choose from different options is also good.” He notes that using PUR can be somewhat more expensive and cumbersome than other methods. For example, although the 13¢ needed to buy a packet of PUR in the Dominican Republic sounds cheap, this may be much more on a per-liter basis than a family would pay for the CDC’s dilute bleach treatment. Plus, the PUR system requires more components—two containers, a stirrer, a filter—than most other systems. The optimal option, Mintz adds, is undoubtedly the kind of built infrastructure found in the developed world.

But Steve Luby, who heads up the CDC’s work in Bangladesh, observes that much of the developing world has waited four or five decades for permanent water treatment systems to arrive. He argues that too many lives are at risk for measures such as PUR to be ignored.

“The numbers [of people at risk] are just huge, and if we wait to build infrastructure we’ll lose a generation,” he says. “We can do something good here, and it also gets people understanding the importance of water and the importance of *clean* water, and the need to actually invest in making water clean. We view this as a step toward community empowerment, toward central infrastructure solutions.”

## Figures and Tables

**Figure:**
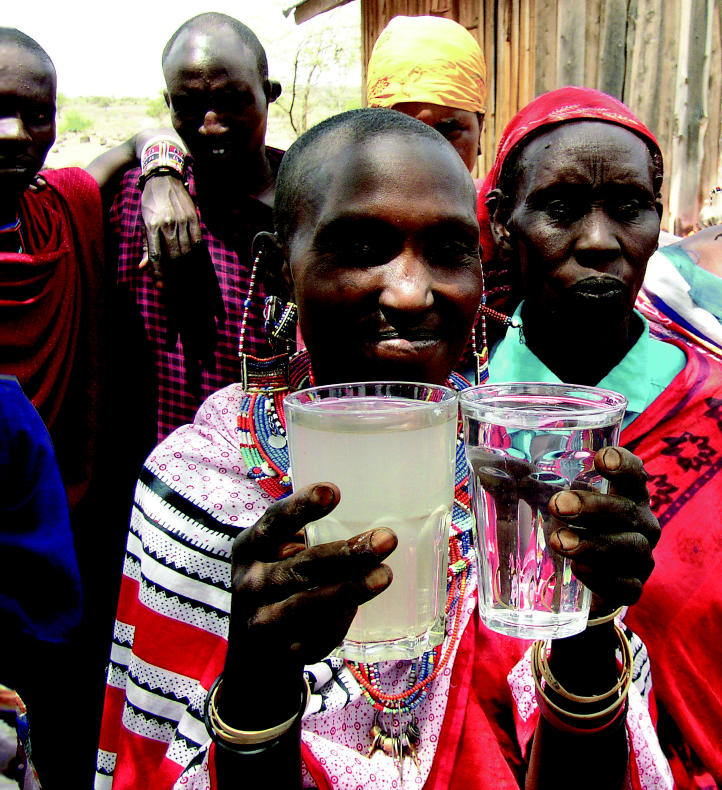
Through a glass clearly A Maasai woman in Kenya holds glasses of polluted water and water treated with a new method to remove contaminants.

**Figure:**
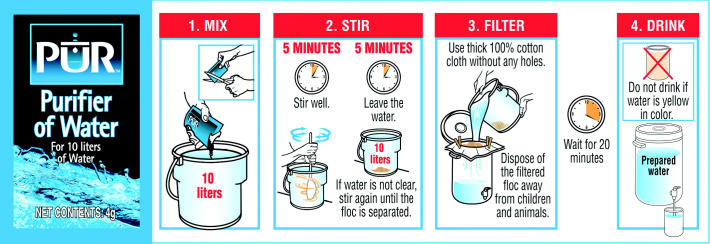


**Figure:**
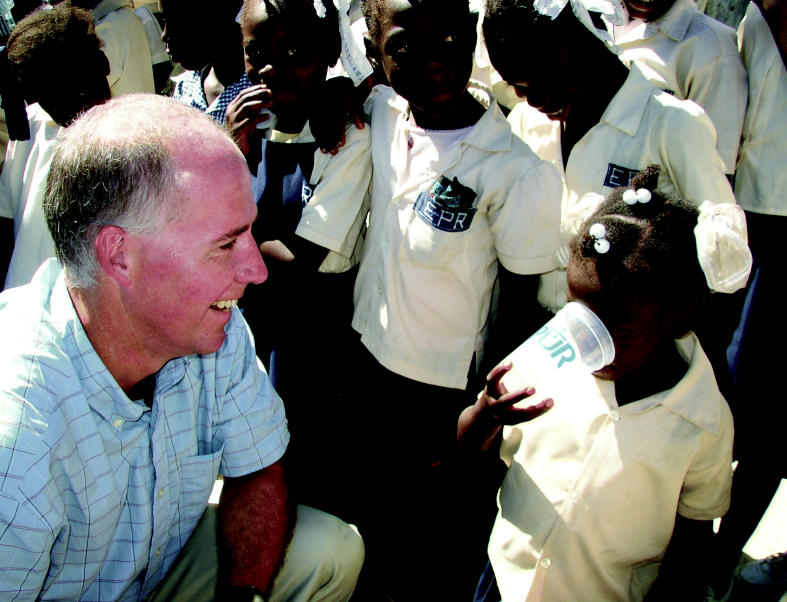
Learning the value of health Greg Allgood (left), developer of the PUR powder, watches as a Haitian schoolchild samples purified water as part of a school outreach program of P&G. The company will invest more than $1 million over the next two years in providing safe drinking water in Haiti’s schools and clinics.
